# Supplementary material for: Causal Relationship between Aging and Anorexia Nervosa: A White-Matter-Microstructure-Mediated Mendelian Randomization Analysis
Source: Biomedicines. 2024 Aug 16;12(8):1874. doi: 10.3390/biomedicines12081874 (PMC11351342; doi:10.3390/biomedicines12081874)
Supplement: Supplementary file 1 [file biomedicines-12-01874-s001.zip › Supplementary Figure/Figure S4/Singe SNP plot of MR analysis for TL to ubm-b-1705.pdf]

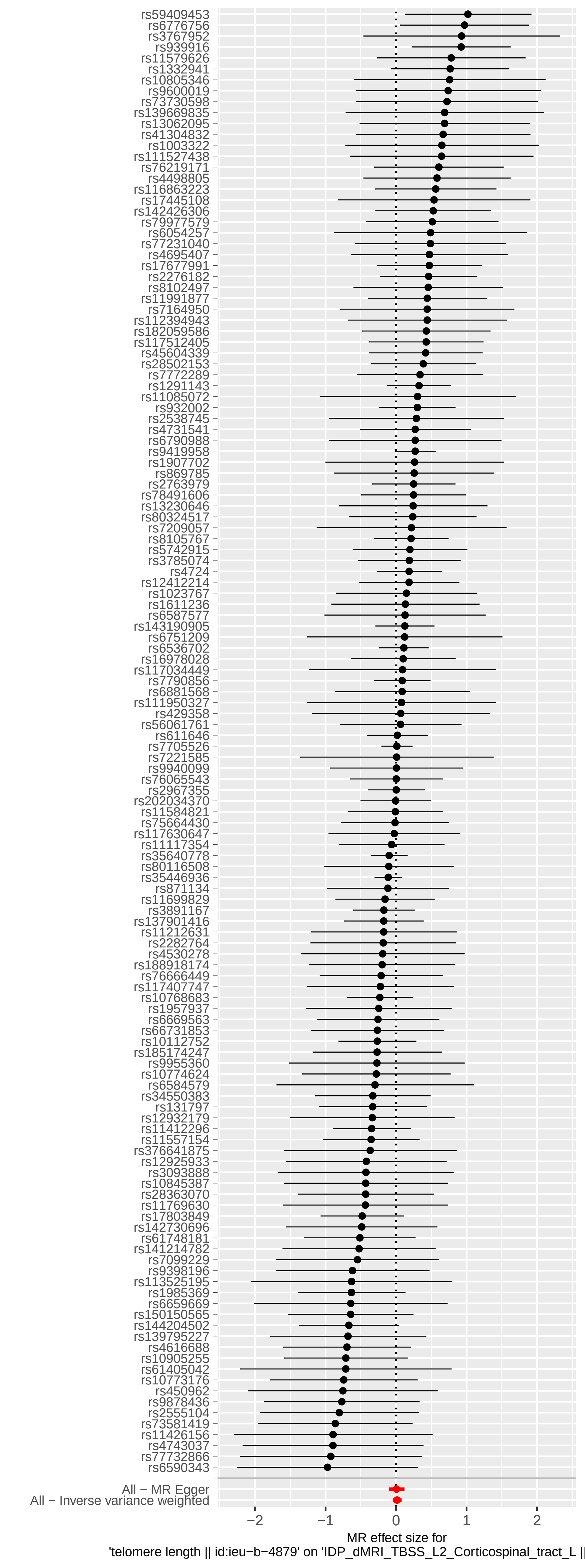

rs59409453
rs6776756
rs3767952
rs939916
rs11579626
rs1332941
rs10805346
rs9600019
rs73730598
rs139669835
rs13062095
rs41304832
rs1003322
rs111527438
rs76219171
rs4498805
rs116863223
rs17445108
rs142426306
rs79977579
rs6054257
rs77231040
rs4695407
rs17677991
rs2276182
rs8102497
rs11991877
rs7164950
rs112394943
rs182059586
rs117512405
rs45604339
rs28502153
rs7772289
rs1291143
rs11085072
rs932002
rs2538745
rs4731541
rs6790988
rs9419958
rs1907702
rs869785
rs2763979
rs78491606
rs13230646
rs80324517
rs7209057
rs8105767
rs5742915
rs3785074
rs4724
rs12412214
rs1023767
rs1611236
rs6587577
rs143190905
rs6751209
rs6536702
rs16978028
rs117034449
rs7790856
rs6881568
rs111950327
rs429358
rs56061761
rs611646
rs7705526
rs7221585
rs9940099
rs76065543
rs2967355
rs202034370
rs11584821
rs75664430
rs117630647
rs11117354
rs35640778
rs80116508
rs35446936
rs871134
rs11699829
rs3891167
rs137901416
rs11212631
rs2282764
rs4530278
rs188918174
rs76666449
rs117407747
rs10768683
rs1957937
rs6669563
rs66731853
rs10112752
rs185174247
rs9955360
rs10774624
rs6584579
rs34550383
rs131797
rs12932179
rs11412296
rs11557154
rs376641875
rs12925933
rs3093888
rs10845387
rs28363070
rs11769630
rs17803849
rs142730696
rs61748181
rs141214782
rs7099229
rs9398196
rs113525195
rs1985369
rs6659669
rs150150565
rs144204502
rs139795227
rs4616688
rs10905255
rs61405042
rs10773176
rs450962
rs9878436
rs2555104
rs73581419
rs11426156
rs4743037
rs77732866
rs6590343
All − MR Egger
All − Inverse variance weighted
−2
−1
0
1
2
MR effect size for
'telomere length || id:ieu−b−4879' on 'IDP_dMRI_TBSS_L2_Corticospinal_tract_L ||
